# Control of signaling molecule range during developmental patterning

**DOI:** 10.1007/s00018-016-2433-5

**Published:** 2016-12-20

**Authors:** Scott G. Wilcockson, Catherine Sutcliffe, Hilary L. Ashe

**Affiliations:** Faculty of Biology, Medicine and Health, University of Manchester, Manchester, M13 9PT UK

**Keywords:** Hh, Wnt, BMP, Dpp, Wg, FGF, Signaling, Cytoneme, stem cell, Embryo, Nodal ECM, Tissue architecture, HSPG

## Abstract

Tissue patterning, through the concerted activity of a small number of signaling pathways, is critical to embryonic development. While patterning can involve signaling between neighbouring cells, in other contexts signals act over greater distances by traversing complex cellular landscapes to instruct the fate of distant cells. In this review, we explore different strategies adopted by cells to modulate signaling molecule range to allow correct patterning. We describe mechanisms for restricting signaling range and highlight how such short-range signaling can be exploited to not only control the fate of adjacent cells, but also to generate graded signaling within a field of cells. Other strategies include modulation of signaling molecule action by tissue architectural properties and the use of cellular membranous structures, such as signaling filopodia and exosomes, to actively deliver signaling ligands to target cells. Signaling filopodia can also be deployed to reach out and collect particular signals, thereby precisely controlling their site of action.

## Introduction

The ability to pattern fields of cells into distinct fates underpins multicellularity. Classical embryology experiments dating back to the early 1900s initially gave rise to the ideas of cell fate induction by other cells or tissues and the existence of gradients of substances that could generate pattern [1, 2]. Spemann and Mangold’s classic experiment revealed that tissue from the dorsal pole of a salamander embryo could induce a secondary axis when transplanted into a recipient embryo, giving rise to the principle of an ‘organizer’ [3]. The term morphogen, or “form producer”, was then later coined by Turing who generated a model to explain how the reaction between these morphogens and their diffusion can generate biological pattern based on their differing concentrations at distinct positions [4]. Various ideas were proposed to explain morphogen gradient establishment and interpretation, including Crick’s source-sink model, whereby localized morphogen production is opposed by distant cells that act as a sink to destroy the morphogen [5], and Gierer and Meinhardt’s activator-inhibitor model, which combines a local self-enhancing activator with a long-range inhibitor activity [6]. Studies such as these offered explanations for the biology that underpins Wolpert’s theory of positional information and interpretation of morphogen concentrations in classical French Flag-type responses [7, 8]. However, it was not until the late 1980s that molecular and genetic studies in *Drosophila* finally enabled the visualization and manipulation of the graded Bicoid and Dorsal proteins that pattern cell fates along the anterior–posterior and dorsal–ventral axes, respectively [9–12]. Although these two gradients are unusual in that they exist in the syncytial embryo, further studies have provided evidence for the gradients of extracellular signals, first for the Bone Morphogenetic Protein (BMP) homologue Decapentaplegic (Dpp) in the *Drosophila* wing and embryo, and gradients of all major classes of signals have now been described [1].

While the simplest mechanism for regulating signaling range is diffusion of a signaling molecule from its source, studies in many contexts have revealed more elaborate mechanisms. In this review, we highlight common themes that have emerged in relation to signaling molecule distribution based on recent studies of different types of signaling molecules in diverse contexts.

## Short-range signaling

In this section, we describe different mechanisms used to generate short-range signaling, showing how local signaling can generate pattern either across a single cell diameter or even within a cellular field.

### Restriction of Dpp diffusion by receptors and co-receptors

**Fig. 1:**
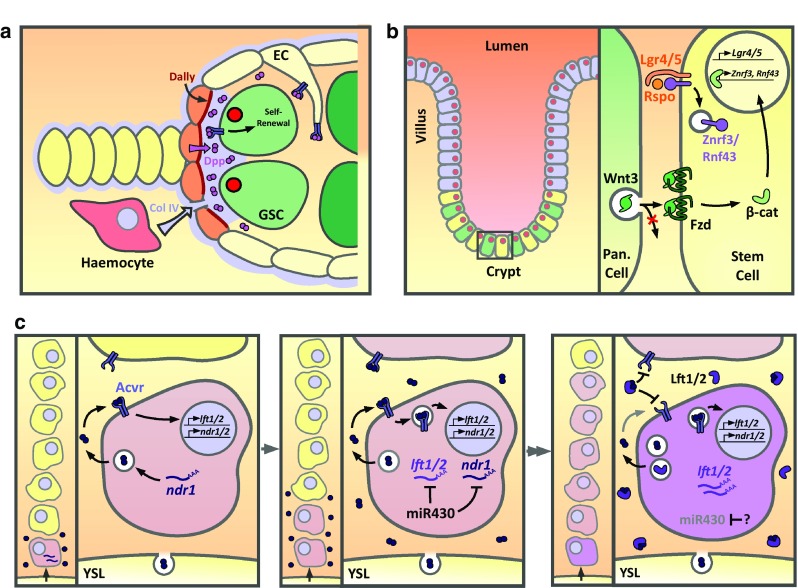
Short-range signaling in tissue patterning. **a** *Drosophila* germline stem cell (GSC) identity is maintained by Dpp secreted by adjacent niche cap cells. Dally, secreted by and presented on cap cells, promotes short-range signaling possibly as a ligand co-receptor. Hemocytes also deposit collagen IV in between cap cells and GSCs, which binds Dpp and restricts its diffusion. Expression of the Tkv receptor on escort cells (EC) acts as a ligand sink. **b** Intestinal stem cell identity (*yellow*) is maintained by neighbouring Paneth cells (*green*) that secrete Wnt3. Wnt3 is bound by Fzd receptors on adjacent cells. Membrane clearance of the ubiquitin ligases Rnf43 and Znf3 is driven by the stem cell factors Lgr4/5 and R-spondin to maintain Fzd levels. **c** *Left panel* maternally loaded *ndr1* in dorsal margin cells and yolk syncytial layer (YSL) *ndr1* expression drives early Nodal signaling (*pink*) in the dorsal-most cells of the presumptive mesoendoderm. *Middle* subsequently a positive feedback loop potentiates the Nodal signal which begins to signal to adjacent cells. The ubiquitously expressed miR430 negatively regulates *ndr1* and *lft1/2* expression. *Right* by the 50% epiboly stage, miR430 expression is lost, enabling *lft1/2* expression which inhibits further activation of Nodal signaling, thereby restricting the domain of responding cells to 5–6 cell tiers. The Nodal signal is maintained over several hours, potentially by signaling from internalised receptors. The cells that receive the signal for the longest (*purple*) have higher levels of pSmad2 resulting in graded signaling

The *Drosophila* ovary is a bundle of ~15 ovarioles, with a germarium structure at the anterior tip of each ovariole. Within the germarium, typically two germline stem cells (GSCs) reside within a niche comprised of somatic cells (Fig. 1a). Upon GSC division, one cell remains as a GSC, while the other daughter exits the niche and differentiates into a cystoblast [13]. Dpp, likely as a Dpp-Glass bottom boat (Gbb) heterodimer, functions as a self-renewal signal acting at exquisitely short-range over only one cell diameter [14]. In this context, the activities of receptors and co-receptors are used to regulate Dpp range and, therefore, GSC number.

Glypicans are a family of heparin sulfate proteoglycans (HSPGs), bound to the outer surface of the plasma membrane via a glycosylphosphatidylinositol (GPI) anchor [15]. The *Drosophila* glypican Division abnormally delayed (Dally) is expressed by niche cap cells and acts within the somatic niche to promote short-range Dpp signaling within GSCs [16, 17] (Fig. 1a). Dally function is limited to cap cells due to repression of *dally* transcription in escort cells (ECs) and escort stem cells that lie posterior to the niche and enclose the germline cells. *dally* repression in these cells is mediated by EGF signaling, with EGF ligands released by germline cells, including GSCs [18]. Removal of Dally from cap cells leads to a loss of GSCs due to differentiation as a result of reduced Dpp signaling, whereas misexpression of *dally* in ECs increases GSC number [16, 17]. In the germarium, Dally function depends on it being membrane localized [16]. Dally binds Dpp [19] and promotes short-range Dpp signaling potentially by concentrating or stabilizing Dpp at the niche, increasing GSC sensitivity to Dpp [16, 17], and/or by acting as a Dpp trans co-receptor, which would limit efficient Dpp signaling to the niche area where Dally on cap cells and BMP receptors on GSCs coincide [17]. It has been proposed that the design of *dally* expression and presentation by niche cells, rather than by GSCs, may facilitate the required loss of Dpp signaling upon cells exiting the niche [16]. In contrast, if the GSCs were to express *dally*, Dpp could remain associated with the cell upon division, which is not compatible with the sharp on–off distinction in Dpp signaling required for the GSC-CB fate change.

In the wing disc, Dally is antagonised by the secreted protein Pentagone (Pent), via an interaction that leads to the internalization and degradation of both Pent and Dally [20]. Pent is itself repressed by Dpp signaling in the wing disc, allowing Dally to enhance local Dpp entrapment and signaling in medial regions of the wing disc [21]. Dpp-dependent repression of *dally* occurs through a conserved silencing element [21] that is functional in GSCs [22, 23]. Therefore, it is likely that repression of *pent* is an important part of the germarium circuitry that establishes the exquisitely short-range Dpp signaling needed to correctly balance GSC self-renewal and differentiation.

In addition to cap cell expressed Dally, expression of the Dpp receptor, Thickveins (Tkv), in ECs appears to restrict Dpp distribution. Wg and Wnt6 expressed by cap cells signal to ECs to directly activate *tkv* expression. Tkv protein is present on EC membranes and projections that extend around GSCs and cysts. Tkv, via its extracellular domain, sequesters Dpp released from the niche to limit the number of GSCs, with GSC expansion detected upon *tkv* knockdown in ECs. In this way the niche has a self-restraining property in that it not only produces the Dpp self-renewal signal but also secretes Wg/Wnt6 that signal to ECs to ultimately restrict Dpp distribution to the niche, thus facilitating differentiation of cells upon niche exit [24]. In addition, cap cells and anterior ECs release Wnt4 that signals within ECs to repress *dpp* transcription. In turn BMPs released from the cap cells appear to attenuate Wnt-responsiveness in anterior ECs, suggesting mutual antagonism between the BMP and Wnt pathways. Interestingly, there is evidence that this BMP-Wnt signaling balance is perturbed as females age, which contributes to the decline in niche function in older females [25]. As described in the next section, there is also a role for the extracellular matrix in regulating BMP signaling range.

### Propagation of Wnt signaling by cell division and regulation of cell surface levels

Generation of graded Wnt signaling by a mechanism that involves regulated receptor turnover and cell division has been proposed in the intestinal stem cell niche [26]. At the base of intestinal crypts, Lgr5^+^ stem cells are maintained in between terminally differentiated niche Paneth cells [27]. Paneth cells produce various signals, including Wnt3, which are required for stem cell maintenance. Single Lgr5^+^ stem cells from mouse crypts can grow into self-organising “mini-gut” epithelial organoids when cultured in vitro in the presence of EGF, Noggin, and the Wnt agonist R-spondin. Lgr5^+^ stem cells initially form symmetric cyst structures, then budding structures that resemble crypts, followed by further expansion to generate the complete organoid. In the organoid multiple crypts, with Paneth cells and stem cells at the crypt base (Fig. 1b), surround a central lumen lined by a villus-like epithelium [28].

Paneth cell-derived Wnt3 is critical for growth of these organoids, and recent visualization of this secreted Wnt3 signal has found it to be enriched on the external surface of Lgr5^+^ stem cells [26]. In this context, the Wnt3 signal appears to not be highly diffusible but instead is mostly found one cell diameter, or occasionally two, away from the Paneth cell source. This signaling requires direct contact between the Paneth and stem cells [26], with previous work suggesting that stem cells maximise their membrane contact with Paneth cells [27]. This limited range of signaling between adjacent cells appears to be due to Wnt binding to its receptor, Frizzled (Fz), which enables the stem cell membrane to act as a reservoir for secreted Wnt3 [26], as had similarly been described in the *Drosophila* embryonic epidermis [29]. Fz is targeted for degradation by the Rnf43 and Znf3 ubiquitin ligases, but this repression can be alleviated by the Lgr5 ligand R-spondin (Fig. 1b). Therefore, Lgr5 signaling leads to membrane clearance of Rnf43 and Znf3 and the maintenance of Fz levels [30, 31]. In addition, propagation of Wnt signaling was shown to require cell division as membrane-bound ligand becomes diluted upon cell division and the inhibition of cell cycle progression restricts the localization of Wnt3 to the producing cells. Together these results show that short-range signaling occurs between Paneth and stem cells and that Fz receptor levels and cell division dictate the signaling range [26]. It remains unclear as to whether or not the transfer of Wnt3 is by passive diffusion that is restricted by high Fz levels or another mechanism. Alternatively Wnt collection by cytonemes (see below) is also a possibility as Lgr4/5 has recently been shown to promote cytoneme formation [32]. It is interesting that the R-spondin/Lgr4/5 signaling module is specific to vertebrates, which may reflect the need to amplify Wnt surface levels on cells that have exited the niche to build up a transit amplifying compartment that can sustain larger vertebrate organ size [26].

A similar process has been suggested to establish a long-range Wnt3a gradient in the mouse paraxial mesoderm [33]. Here, Wnt3a expression is restricted to the posterior presomitic mesoderm and ligand production is proposed to cease as cells exit the tail bud. Based on β-catenin nuclear localization and target gene expression, a gradient of Wnt activity forms; however, Wnt localization has not been observed in this context. One possibility is that Wnt gradient formation occurs through the inheritance and dilution of receptor-bound and/or intracellular ligand [33]. An alternative explanation could be that cells that exit the tail bud retain transcriptional memory of earlier signaling, as has been suggested in the *Drosophila* wing disc (see below).

### Establishment of graded Nodal signaling by autoactivation and timed inhibition

In the zebrafish embryo, cells at the margin are fated to become mesendoderm by Nodal signaling via the Nodal-related ligands, Ndr1 (also called Squint) and Ndr2 (also known as Cyclops). Here, a temporal window for signal activation defines the spatial dimensions of the Nodal signaling domain in a mechanism involving differences in timing of the production of Ndr1/2 and their antagonists Lefty1 and 2 (Lft1/2), primarily due to miRNA repression of the latter [34]. Nodal signaling, dependent on maternal *ndr1* expression, initiates in the dorsal-most cells at the sphere stage before Nodal signaling is then activated within all cells at the margin by zygotic *ndr1/2* expression in the yolk syncytial layer. Ndr1/2 autoactivates so that, over time, Nodal signaling is progressively transcriptionally activated in the next tier of adjacent cells through this positive feedback, which results in spreading of the Nodal signaling domain in the direction of the animal pole (Fig. 1c). Expression of the *lft1/2* antagonists is also activated by Nodal signaling in these cells; however, the maternal *ndr1* expression and production of Ndr1/2 by the yolk syncytial layer permit initiation of Ndr1/2 signaling prior to *lft1/2* transcription. More importantly, the *lft1/2* mRNAs are translationally repressed by members of the miR-430 family, which are initially ubiquitously expressed. This miR-430-mediated temporal delay in Lft1/2 protein accumulation allows the Nodal signaling domain to spread spatially, until a threshold of Lft1/2 is reached that is sufficient to inhibit Ndr1/2 signaling [34]. The miR-430 family also represses the *ndr1* mRNA, but not *ndr2* [35].

Lft1/2 proteins eventually accumulate following a loss of *miR*-*430* repression, through an as yet unknown mechanism, at the 50% epiboly stage. Lft1/2 proteins inhibit Ndr1/2 signaling to prevent further expansion of the signaling domain, which is limited spatially to 5–6 tiers of marginal cells. However, signaling within the domain of cells that received Ndr1/2 ligands persists for several hours, potentially due to signaling from internalized receptors. Moreover, within the Nodal signaling domain, dorsal margin cells that have received Ndr1/2 signals for a longer duration have higher pSmad2 levels resulting in graded signaling across the active domain [35]. There is evidence that Ndr2 is particularly important in this region for the extended duration of signaling [36]. Ndr2 is less stable than Ndr1 based on measurements in tissue culture cells, due to the presence of a lysosomal targeting domain in Ndr2. As this differential stability can restrict signaling range when Ndr1 and Ndr2 are misexpressed in zebrafish embryos [37], the instability of Ndr2 may also contribute to its high-level, short-range signaling in dorsal margin cells.

Overall, this graded Ndr1/2 signaling results in the highest levels of pSmad2 and downstream transcriptional targets in a dorsal-to-ventral gradient at the blastula stage, then in a vegetal-to-animal gradient in late blastula stage embryos. Restriction of Ndr1/2 signaling to the six cell layers can be reconciled with the previous reports suggesting the expression of long-range Nodal targets beyond this domain, as these genes were instead found to be activated by FGF signaling, which itself is activated by Ndr1/2 signaling [34]. Finally, temporal control of Nodal receptor activation using an optogenetic approach has revealed how an extended duration of Ndr1/2 signaling in the organizer is interpreted at the gene expression level to promote prechordal plate specification and suppress endoderm differentiation [36].

## Tissue architecture

The examples in this section describe how tissue architecture can modify signaling molecule distribution and activity at various levels, from composition of the local extracellular matrix environment through to tissue macro-structure.

### ECM-sequestration of BMP

As described above, Dally and Tkv, expressed in the germarial niche and ECs, respectively, play a role in restricting Dpp distribution and signaling to GSCs. However, another mechanism, involving the collagen IV extracellular matrix protein, also limits Dpp protein to the ovarian GSC niche [38, 39]. In *Drosophila*, collagen IV is encoded by the *viking* and *Dcg1* genes [40]. Collagen IV is present within a basement membrane (BM) lining the germarium, from which projections extend into the region between the cap cells and GSCs, forming a specialized BM within the GSC niche [38, 39]. Niche collagen IV is not deposited by germarium cells, but instead by plasmatocytes, a type of hemocyte (blood cell) (Fig. 1a). Plasmatocytes associate with the larval gonad and build a specialized BM during niche differentiation that remains stable throughout adult life [39]. This BM is required for GSC niche homeostasis, as extra GSCs are observed in germaria from *viking* hypomorphic mutant females with reduced collagen IV levels [38, 39], upon hemocyte-specific knockdown of either collagen IV or an enzyme required for collagen IV biosynthesis, or by ablation of the hemocytes [39]. Dpp has been shown to bind to the non-collagenous C-terminal domain of collagen IV [38, 41]; therefore, together, these results suggest that niche collagen IV sequesters Dpp to limit its signaling range and GSC number [38, 39]. In support of this, the additional GSCs observed in germaria from females with hemocyte-specific collagen IV knockdown express the Dpp signaling reporter *Dad*-*LacZ* and the extra GSC phenotype can be rescued by removal of a single copy of *dpp* [39].

It will be interesting to investigate the interplay between Dally, collagen IV, and EC-expressed Tkv in regulating Dpp distribution/signaling through analysis of double mutants, as currently it is unclear, for example, why collagen IV and Tkv cannot concentrate Dpp in *dally* mutants to retain GSCs. While the focus here has been on regulation of Dpp distribution, multiple mechanisms also exist in the cystoblast to extinguish transduction of the self-renewal Dpp signal within this cell that is destined for differentiation [13].

### An ECM scaffold directs BMP gradient formation

In the *Drosophila* early embryo, collagen IV proteins play a key role in the generation of the BMP gradient that patterns cell fates in the dorsal ectoderm, but in this case collagen IV has a scaffold rather than barrier function [38, 41, 42]. In the embryo, the most potent BMP is a Dpp–Screw (Scw) heterodimer. As *dpp* mRNA is uniformly expressed in the dorsal ectoderm and *scw* expression is ubiquitous in the embryo, formation of the Dpp–Scw gradient involves redistributing the heterodimer across a field of dorsal ectoderm cells that uniformly express it. This is achieved by two extracellular BMP-binding proteins, Short Gastrulation (Sog) and Twisted Gastrulation (Tsg), that form an inhibitory complex with Dpp–Scw, as well as a protease, Tolloid (Tld), which cleaves Sog within this complex to liberate Dpp–Scw. *tsg* and *tld*, like *dpp*, are expressed in the dorsal ectoderm, whereas *sog* is expressed in the neuroectoderm underlying the dorsal ectoderm [43].

**Fig. 2:**
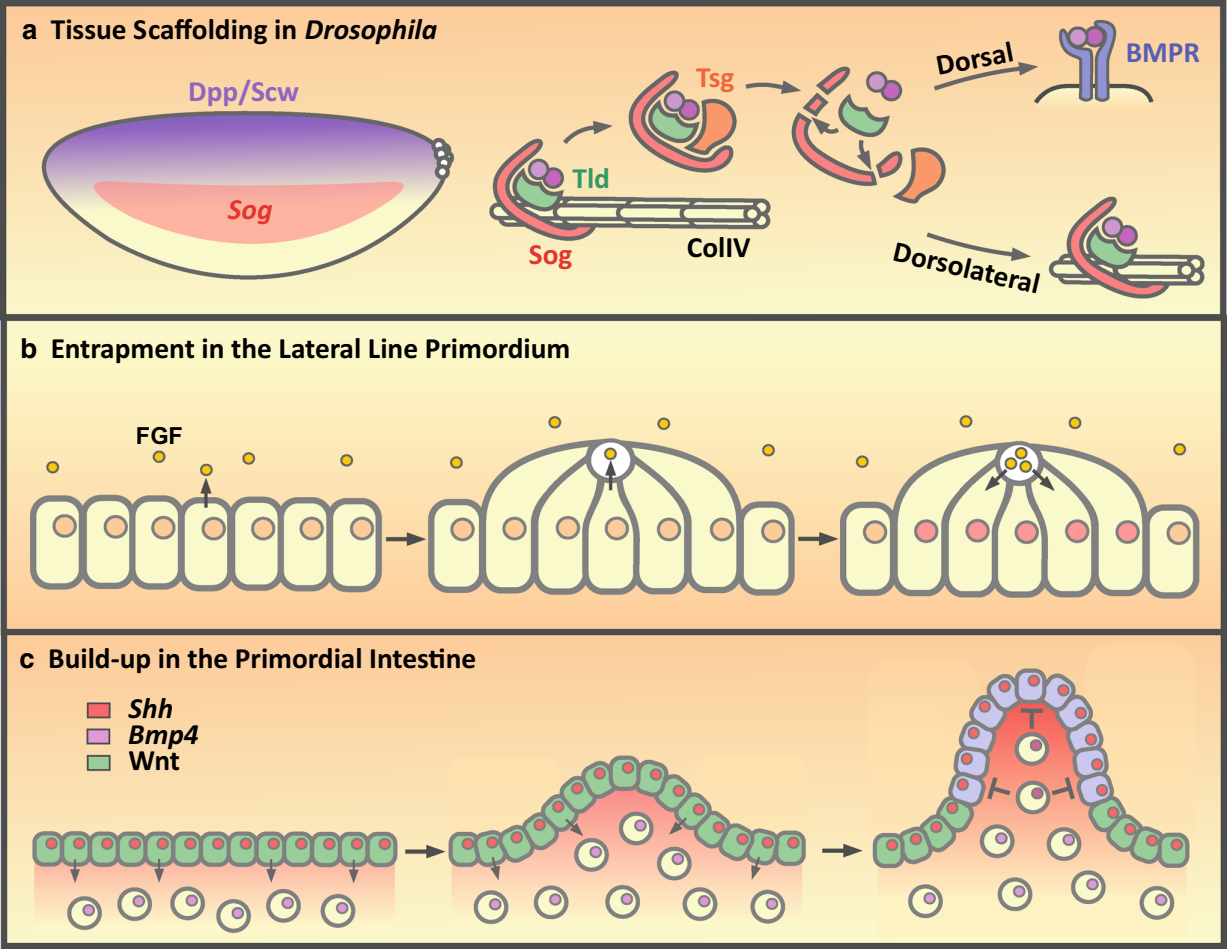
Influence of tissue architecture on signaling range. **a** Dorsally secreted Dpp–Scw heterodimers bind to collagen IV along with Sog and Tld. Inhibitory complex formation on Collagen IV (ColIV) acts to restrict dorsolateral signaling but also enables release of a freely diffusing shuttling complex by Tsg. Proteolytic cleavage of Sog by Tld releases the Dpp–Scw heterodimer which, if dorsally localized, can promote Dpp signaling. However, if cleavage occurs dorsolaterally, then the inhibitory complex is reformed. **b** *Left panel* the zebrafish lateral line epithelial primordium migrates along the flanks of the fish and secretes FGF ligands. *Middle* apical constriction of the epithelial cells drives the formation of a ‘rosette’ shaped organ with a central microlumen into which FGF is secreted. *Right* after FGF levels reach a threshold, the cells stop migrating and continue mechanosensory organ development. **c** *Left panel* the early chick gut epithelium (*green*) retains a stem cell-like identity (Lgr^+^, Sox9^+^) in response to Wnt signaling and secretes Shh to drive low-level *Bmp4* expression in the underlying mesenchyme. *Middle* mechanical stress later results in the buckling of the epithelium. *Right* the concentration of Shh at the tip of the folds results in increased *Bmp4* expression in the fold mesenchyme. BMP4 signals back to the gut epithelium to repress Wnt signaling to promote the differentiation of the fold epithelium, or villus, and restrict the stem cell pool to those cells at the base of the fold. For simplicity, the cytoplasmic colour is used to depict the stem cell or differentiated fates, depending on the presence or absence of Wnt signaling, respectively, whereas the nuclear colour depicts expression of Shh or Bmp4

Embryos with lower collagen IV levels show disrupted Dpp–Scw signaling with a reduced gradient. As Dpp, Sog and Tld bind to collagen IV, it has been proposed that collagen IV acts as a scaffold for assembly of a Dpp–Scw–Sog–Tld–Tsg shuttling complex [38, 41, 42] that is essential for gradient formation [43] (Fig. 2a). In this model, following secretion, Dpp–Scw, Sog and Tld all bind independently to the collagen IV extracellular matrix [38, 41, 42]. Remodeling of protein interactions allows these proteins to interact with each other on collagen IV. Tsg cannot bind collagen IV [38], but instead releases Dpp–Scw–Sog–Tld from collagen IV as a freely diffusing Dpp–Scw–Sog–Tld–Tsg complex [38, 41, 42] (Fig. 2a). In this model, BMP gradient formation depends on a balance between how far the Dpp–Scw–Sog–Tld–Tsg complex can diffuse and its rate of cleavage by Tld. If Tld cleavage occurs in dorsolateral regions of the embryo, where levels of neuroectodermal-derived Sog are high, the inhibitory complex will reform on collagen IV (Fig. 2a). However, if the Dpp–Scw–Sog–Tsg–Tld complex has diffused near the dorsal midline, when Tld cleavage occurs the lack of Sog protein in this region will allow Dpp–Scw to bind its receptors and signal.

Overall, this model provides an explanation for the observation that Dpp cannot diffuse in the embryo in the absence of Sog and Tsg [44], as here both are required to release Dpp–Scw immobilized on collagen IV [38, 41]. Moreover, a scaffold role for collagen IV is supported by whole-organism modeling data, which reveals that the binding affinity between Sog (or the vertebrate ortholog Chordin) and BMP is too low for the rate of complex formation required in vivo. However, the modeling data are a good fit to the in vivo data when a scaffold is included that reduces Sog and BMP diffusion, locally increasing the Sog and BMP concentrations and facilitating their interaction [45].

The mechanism of DV patterning is conserved in vertebrates, including *Xenopus* embryos where the activity of BMPs is counteracted by antagonists such as Chordin that are released from Spemann’s organizer. One minor difference is that Tolloid proteases, predominantly BMP-1, degrade free Chordin, unlike Tld cleavage of *Drosophila* Sog that only occurs when Sog is bound to BMP [46, 47]. In *Xenopus,* the BMP gradient is stabilized by the dorsally expressed ONT-1 extracellular matrix protein. ONT-1 binds Chordin and BMP-1/Tolloid proteases, which promotes their association and, therefore, Chordin degradation [48]. In addition, a long-range Chordin gradient has recently been visualized in Brachet’s cleft, a narrow region rich in fibronectin extracellular matrix that separates the ectoderm from the mesodermal and anterior endodermal layers. Overexpressed BMPs are also detected within Brachet’s cleft, and knockdown of BMP-1 increases Chordin levels in Brachet’s cleft, particularly ventrally, with the opposite effect observed upon knockdown of the Chordin stabilizer Sizzled. It has been suggested that this single Chordin-BMP gradient may pattern the ectoderm and mesoderm through cell contact or proximity to the cleft, from which Chordin-BMP signals can be released [49]. As both Chordin and BMP-1 have been reported to bind fibronectin [50], it will be interesting to determine whether the fibronectin ECM in the cleft has a scaffold like property, akin to that of collagen IV in the *Drosophila* embryo.

### Tissue morphogenesis drives local ligand entrapment

Distinct from this role of the local extracellular environment in modulating BMP distribution, recent studies highlight the role of tissue architecture and its dynamic changes throughout development in regulating cell fate decisions. Development of the zebrafish posterior lateral line system involves the assembly and sequential deposition of mechanosensory organs by a collectively migrating epithelial primordium along the flanks of the embryo [51]. FGF ligands, expressed by the epithelial cells, play a fundamental role in this process by arresting cell migration through the regulation of chemokine receptor (*cxcr4b* and -*7b*) expression [52] and controlling deposition and epithelialisation to generate a stereotypical ‘rosette’ organ structure [53, 54]. Here, the regulation of ligand diffusion is coupled to organ morphogenesis as Fgf3 is trapped within a lumenal structure (Fig. 2b).

Time-lapse imaging of developing embryos reveals that the pattern of organ spacing is determined by the timing of organ deposition in response to Fgf3, rather than the speed of the collective cell migration or embryonic growth. By inhibiting FGF ligand activity, the timing of organ deposition is delayed and organs are widely spaced. Conversely ectopic expression of Fgf3-GFP expression results in early organ deposition and organ spacing is reduced in a concentration-dependent manner [51].

Imaging of uniformly overexpressed Fgf3-GFP reveals strong localization within spherical microlumina that form through the apical constriction of organ epithelial cells [46]. All cells of the organ progenitor can be seen to share contact with the microlumina at the apical centre with tight and adherens junctions characteristic of a lumen. Photobleaching and recovery of Fgf3-GFP show that Fgf3 is mobile within the microlumen. Fgf3-GFP’s microlumenal accumulation can also be blocked by the protein secretion inhibitor Brefeldin A, which results in Fgf3-GFP accumulation intracellularly within vesicular structures in epithelial cells. Together these results illustrate that Fgf3 localization and diffusion are highly restricted in this tissue. Clonal expression of Fgf3-GFP by small populations of organ progenitor cells only affected the deposition timing of the mosaic organs and not their neighbouring wild-type organs. This suggests that FGF signaling is not acting as a long-range morphogen in the regulation of organ deposition, but is instead acting locally within the microlumen and affecting only those cells that are attached. Indeed, disrupting the formation of the microlumen, either through the knockdown of the actin-binding Shroom3 which is required for apical organ epithelial constriction or through two photon laser micropuncture, results in Fgf3-GFP leakage and a delay in organ deposition due to prolonged migration. These findings show the importance of this microenvironment in the regulation of FGF signaling in the development of the embryonic nervous system. Following micropuncture the epithelium eventually recovered and reformed the lumen illustrating their dynamic and plastic nature. The authors propose a model whereby the formation of the microlumina and subsequent entrapment of secreted FGF ligands act a timer that coordinates organogenesis and organ deposition within the developing embryo [51]. Lumen formation is likely a conserved and common method of regulating organogenesis as similar structures can be seen to form dynamically throughout organismal development as well as being a characteristic activity of self-organising complex organoids [55–57].

### Tissue morphogenesis directs patterning centre formation

A similar method of coupling tissue morphogenesis with the regulation of cell fate was recently described in the mouse and chick developing gut [58]. The primordial gut and early gut epithelium constitutes a stem cell-like pool based on the uniform expression of the adult intestinal stem cell (ISC) marker Lgr5-GFP and the Wnt signaling target gene *Sox9* [58–60]. During gut development, the expression of ISC markers, and therefore stem cell fate, becomes increasingly restricted in conjunction with proliferative rate until finally being restricted to those cells between the villi. Here, uniformly secreted ligands form discrete signaling centers as a direct result of the changing morphology of the primordial gut. This enables uniformly expressed morphogens to pattern the gut epithelium and underlying mesenchyme to restrict the initially uniform stem cell population.

It has previously been shown in chick embryos that Shh is expressed in the primordial gut and induces expression of *Bmp4* in the underlying mesenchyme [58, 61]. Inhibition of either Shh or BMP signaling through the application of cyclopamine or Noggin, respectively, results in an increase in endodermal proliferation [58]. However, application of both Shh and Noggin also results in increased proliferation, consistent with Shh-dependent mesenchymal induction of *Bmp4* expression being necessary for the repression of endodermal proliferation. In addition, cyclopamine treatment also results in the maintenance of uniform *Lgr5* expression throughout the gut showing that this reciprocal signaling cascade is necessary for the restriction of ISC numbers. This is achieved by the BMP-dependent repression of Wnt signaling, illustrated by the repression of *Sox9* expression in gut explants cultured in the presence of Shh or BMP4. This model describes how adult ISCs are specified but how do uniformly secreted morphogens define discrete stem cell populations? The answer comes from the tissue architectural changes that occur in the developing gut. Due to sequential compressive forces generated by the underlying smooth muscle, the primitive gut epithelium begins to buckle and form ridges that eventually form into villi. The authors illustrate this idea through computational modeling of an epithelium uniformly expressing a ligand. As the epithelium becomes increasingly folded, as is seen in the developing gut, ligand concentration can be seen to increase significantly within the folds as the number of ligand-producing cells concentrates at the tip of the fold (Fig. 2c). This model enables the generation of a discretely localized mesenchymal signal (BMP4) from a uniform endodermal signal (Shh). Indeed, when Shh localization is visualized in the chick gut at embryonic day 13 (E13), when the epithelial folds are still broad, there is low-level diffusion of Shh into the fold mesenchyme. By E15, when the villi are taking shape, Shh is concentrated within the fold mesenchyme and strongly induces BMP4 expression which, in turn, represses Wnt signaling in the overlying endoderm [58] (Fig. 2c).

Manipulation of the tissue mechanics shows that these folds are both necessary and sufficient for primitive gut patterning. The authors sliced mouse and chick primitive gut tubes to generate ringlets that when cultured for 36 h continue to develop villi through the constriction of the outer smooth muscle. However, if the ringlets are inverted, the outer endoderm and mesenchyme are unable to fold to the same degree and *Bmp4* and *Sox9* expression remains uniform. Conversely, the control ringlets display the stereotypical folding and signaling to define the ISC population, showing that gut folding is necessary for pattern formation. The reciprocal experiment involved the culturing of early explant primitive guts under a fine grid. As the tissue grows over 36 h into the grid, small pseudo-villi form long before endogenous villi. Here, uniform Shh expression is induced throughout the pseudo-villi endoderm, but areas that were highly curved displayed high levels of *Bmp4* expression and phospho-SMAD staining as well as decreased *Sox9* expression and proliferation in comparison with explants not grown under a grid. This experiment shows that primitive gut folding is sufficient for the patterning of the gut endoderm in mice and chick. Through these morphological changes during embryonic gut organogenesis, the ISC population is limited to those cells between the villi at their base [58].

## Membranous protrusions

It has long been established that primary cilia, microtubule-based cellular protrusions, act as essential signaling platforms for Hh signaling [62] and more recently have been identified as regulators of Notch signaling in eye development [63]. In this section, we describe the role of long membranous processes, distinct from cilia, in the regulation of morphogen signaling range.

### Nanotubes promote BMP signal transduction

Microtubule-based (MT-) nanotubes (further reviewed in [64]) have been identified as a type of cytoplasmic protrusion involved in restricting the range of morphogen gradients in the *Drosophila* testis [65]. While they bear a striking resemblance to primary cilia, they have been suggested to represent a distinct cellular organelle characterized by their lack of microtubule acetylation, sensitivity to fixation, and the frequent absence of any association with the basal body [65]. In this context, MT-nanotubes are proposed to act as a signaling platform for short-range Dpp signaling in GSC maintenance.

These protrusions were visualized through the expression of GFP-tagged α-tubulin and were found to be generated by GSCs and extend in a largely uniform orientation into the stem cell niche hub cells [65]. In contrast, MT-nanotubes are only very rarely found on differentiating cells where they are non-uniformly orientated. MT-nanotubes are absent from mitotic GSCs, with GSCs forming MT-nanotubes upon their exit from mitosis. RNAi knock down of components of the intraflagellar transport-B complex that are necessary for primary cilia assembly and function reveals a similar requirement for MT-nanotube formation as they become significantly shorter and form less frequently. However, proteins involved in cytoneme formation, such as Diaphanous (see below), are not required for MT-nanotube formation [65].

To determine the role of MT-nanotubes in hub-GSC signaling, the localization of Dpp signaling components was visualized, as Dpp acts as a critical GSC maintenance signal [66]. Live imaging revealed that fluorescently tagged Tkv is trafficked from germ cells in puncta along MT-nanotubes extending into the hub [65]. Moreover, Dpp expressed from the hub cells co-localizes with Tkv expressed by germline cells, suggesting that Dpp–Tkv interaction occurs at the MT-nanotube surface–hub cell plasma membrane interface. Disruption of MT-nanotube formation decreased the number of Tkv-puncta in the hub area and compromised Dpp signaling, resulting in GSC loss when tested as mutant clones in competition with wild-type GSCs. In contrast to Tkv, the Unpaired receptor of JAK-STAT signaling, another pathway important for GSC maintenance [66], was observed within GSCs and not in MT-nanotubes, revealing that MT-nanotubes show specificity for the proteins that they traffic [65].

Furthermore, manipulation of the Dpp pathway reveals Dpp–Tkv interaction to be both necessary and sufficient for MT-nanotube formation. While shorter, less frequent MT-nanotubes were observed in a *dpp* mutant or upon germline knockdown of *tkv*, overexpression of *tkv* resulted in longer MT-nanotubes. Expression of a dominant negative form of Tkv carrying the extracellular domain but lacking the intracellular domains resulted in the thickening of MT-nanotubes, demonstrating that ligand–receptor interaction, and not downstream signaling is important for MT-nanotube formation [65]. In addition, overexpression of *dpp* in somatic cyst cells resulted in the formation of ectopic MT-nanotubes. Overall, it was proposed that MT-nanotubes contribute to short-range Dpp signaling by forming a specialized cell surface area for productive Dpp–Tkv interactions that selectively allow GSCs, but not gonialblasts, to access the high concentration of the self-renewal Dpp signal in the niche [65].

### Reaching out for ligands

Cytonemes (‘cell threads’) are specialized actin-based signaling filopodia, ~0.2 µm in diameter, and of varying lengths reaching up to 80 µm [67] that are found to orient and extend away from the cell body toward distant signaling centers [68]. Recently studies have begun to elucidate the role of cytonemes in a process reminiscent of that seen in neuronal signaling (as reviewed in [69]).

**Fig. 3:**
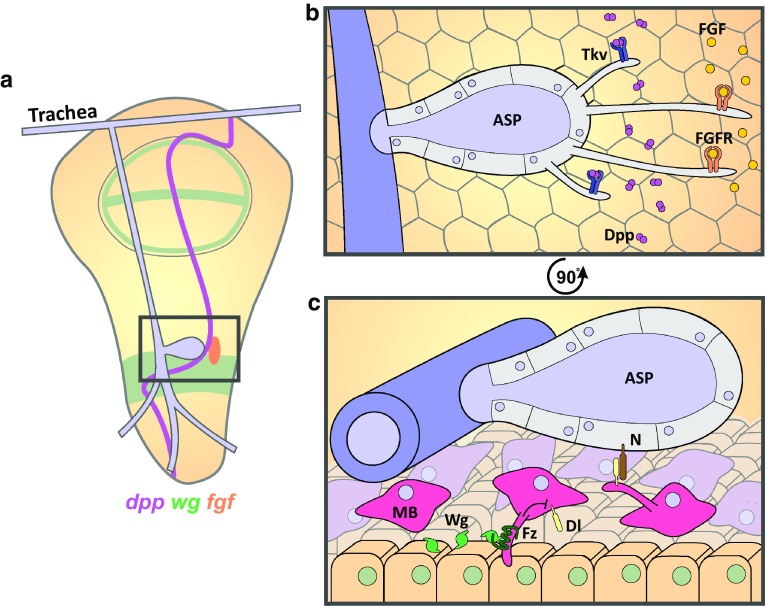
Cytonemes deliver and reach out for ligands in Air Sac Primordium development. **a** Schematic of the wing disc in third instar *Drosophila* larvae showing the tracheal branch, bound to the wing disc, with the ASP budding from the transverse connective (*grey box*) in response to the morphogens Dpp (*purple*, A/P border), Wg (*green*), and FGF (*orange*). **b** Enlarged view of the *box* in **a** showing that cells of the medial ASP (*purple*) extend short Tkv-loaded cytonemes and long FGFR-loaded cytonemes from the tip to capture distant Dpp and FGF secreted by cells of the wing disc epithelium. **c** Magnified, 90 °C rotated view in **b** showing that myoblasts underlying the ASP extend Fz-loaded cytonemes toward Wg-expressing cells, activating signaling to inhibit Dl expression. In turn, myoblasts extend cytonemes carrying Dl to the ASP, activating Notch signaling to promote correct ASP development

Reciprocal signaling between the cells of the wing disc epithelium; overlying flight muscle progenitors (myoblasts), and tracheal epithelial cells directs the formation of the Air Sac Primordium (ASP). This epithelial monolayer surrounds an air-filled lumen and will eventually form the dorsal air sac [70–72]. Budding of the ASP from the transverse connective branch of the trachea, overlying the wing disc, is regulated by morphogens secreted from the disc epithelium. The tracheal patterning FGF ligand, Branchless, is secreted by a small subset of posterior disc cells and promotes ASP growth and migration [71], while Dpp is expressed in a stripe along the A/P border forming a graded band of signal transduction [73, 74] that is necessary for ASP development [75] (Fig. 3a). Driving tracheal expression of CD8-GFP similarly reveals ASP derived cytonemes that extend toward the secreted pools of Dpp and FGF [71, 75] (Fig. 3b, c). Intriguingly, the apical ASP cells were found to generate two distinct types of cytoneme that are induced by either Dpp or FGF signaling. Dpp-induced cytonemes were found to be relatively short (2–15 µm) and extend distally from the lateral flank of the ASP to directly contact Dpp-producing cells. Fluorescently tagged-Dpp is taken up by Tkv-positive cytonemes and can be visualized within motile puncta [75]. FGF-induced cytonemes are generally longer (12–50 µm) and extend from the ASP tip toward the producing cells and express the FGF receptor Breathless [75] (Fig. 3b).

Various factors were identified as regulators of cytoneme formation. Perturbation of the function of the formin Diaphanous, the adhesion molecule Neuroglian or Dynamin (Shibire) caused a reduction in cytoneme number and length. This resulted in a decrease in ASP-Dpp signaling and caused malformation of the ASP. In addition, loss-of-function of the adhesion molecule Capricious also reduced the ability of cytonemes to contact Dpp-producing cells and caused abnormal ASP development [75]. The authors conclude that cytoneme-mediated uptake of Dpp and FGF is essential for signal activation within the ASP to drive its development [75]. In addition, the ECM plays a role in cytoneme extension [76]. Dally and Dlp are necessary for Dpp and FGF responsive cytoneme migration, respectively. Cytonemes are unable to migrate across the respective mosaic mutant clones within the wing disc, leading to a reduction in signal activation and small, abnormally shaped ASPs. The secretion of these HSPGs by disc cells, but not their expression, is regulated by the planar cell polarity proteins Van Gogh and Prickle, although the mechanism has yet to be elucidated. Another ECM component, laminin, was also reduced in *prickle* mutant clones and loss of the integrin subunits αPS1, αPS2, and βPS, and Integrin-linked kinase resulted in abnormal ASPs and a reduced number of cytonemes. Furthermore, while flies heterozygous for mutations in a laminin subunit or αPS1 develop normal ASPs, those of the double heterozygous flies developed abnormally with fewer cytonemes and reduced Dpp and FGF signal transduction. Together these results suggest that laminin-activated integrins are also necessary for cytoneme function, while Dally and Dlp act as substrates for cytoneme growth [76].

Cytonemes have also been suggested to provide a means for the indirect effects of Wingless (Wg) on ASP development through myoblast-mediated Notch signaling [77]. Notch is required for ASP development with loss-of-function mutants leading to severe reduction in ASP size [77]. Signal activation is sensitive to the levels of the ligand Delta (Dl) expressed in the flight muscle progenitors, or myoblasts, which lie between the ASP and disc epithelium [77, 78] (Fig. 3c). Visualization of myoblast-derived Dl localization reveals motile puncta within cytonemes travelling at 0.33 µm/s toward the ASP, a speed consistent with myosin motor driven transport. These cytonemes contact tracheal cells expressing Notch and enable signal activation necessary for ASP development [77].

Interestingly, Wg overexpression in the wing disc phenocopies *Notch* loss of function, causing a severe inhibition of ASP development despite the absence of Wg signaling within the ASP [77]. The use of GFP reconstitution across synaptic partners (or GRASP), whereby fragments of GFP are expressed by distinct cellular populations and only fluoresce when the two components are in close proximity (<100 nm) [79], revealed that the overlying myoblasts form cytoneme-mediated contacts with Wg-producing disc cells during the early stages of the third instar of larval development (Fig. 3c). These cytonemes present the Wg receptor Frizzled and internalize the ligand, which can be seen in vesicular structures within the cytonemes. Wg signaling down-regulates Dl levels within the myoblasts, enabling the indirect regulation of Notch signaling by the disc cells. It was suggested that individual myoblasts may have cytonemes extending toward both the wing disc and the ASP. As larval development progresses, the distance between the myoblasts contacting Wg-producing cells and the myoblasts contacting the ASP increases, suggesting that Wg signaling is only relevant to the ASP in the early larval development to control Dl-dependent Notch signaling [77].

Similar but distinct structures have also been described in vertebrates. Airinemes are cellular projections that were identified in zebrafish xanthoblasts, non-terminally differentiated neural crest cells, and predicted to have a role in Notch signaling during pigmentation. Xanthoblasts are suggested to extend Dl positive airinemes that contact Notch-expressing melanophores, promoting Notch signaling to increase melanophore stripe formation. Although airinemes show some similarities to actin-based cytonemes, airinemes are also dependent on microtubules and exhibit more convoluted trajectories, move faster and are associated with larger exosome-like vesicular particles [80].

In the chick limb bud, Filopodia-Like Cellular Protrusions (FiLiPs) are produced by the epithelial cells of the dermomyotome and are proposed to play a role in somite development [81]. They connect the epithelial somites to the overlying dorsal surface ectoderm spanning the subectodermal space. Like airinemes, FiLiPs are both actin- and microtubule-based structures and were shown to be regulated by Cofilin, Fascilin, microtubule motor proteins, and dependent on Rac1 for their formation. They contain the Wnt receptor Frizzled7, moving in punctae with a net retrograde motion and are proposed to mediate long-range paracrine Wnt signaling during limb bud development [81].

### Ligand delivery

The *Drosophila* ovarian stem cell niche highlights a different functionality of filopodia-like protrusions. As previously described, niche somatic cells maintain GSC self-renewal through the secretion of the BMP ligands Dpp and Gbb [82]. The expression of *dpp* and *gbb* in ECs is regulated by niche Cap cells (CpCs) that express *hh* and have been shown to transport Hh to ECs along short cytonemes [83, 84]. These cytonemes differ from those identified in the wing disc as they grow from ligand-producing cells to deliver the Hh ligand to receiving cells, resulting in upregulation of BMP ligand expression in ECs [83, 84]. The cytonemes also allow the niche to respond dynamically to changes in Hh levels. When Hh signaling is lowered in ECs, the cytonemes extend up to sixfold longer than those found under homeostatic conditions, projecting towards signaling-deficient areas of the niche to increase Hh spreading range [84].

This same mechanism of ligand delivery has also been visualized in a number of developmental contexts. In the chick limb bud, Shh is produced by mesenchymal cells at the Zone of Polarizing Activity (ZPA). From there it acts over a long-range to specify digit identity and is able to act far beyond its site of production [85, 86]. Actin-based filopodia-like protrusions have been identified as extending from the mesenchymal cells. They can be seen to span several cell diameters, extending away from the ZPA in a net apical direction and are suggested to direct long-range transport of Shh. Shh puncta are seen to travel along these protrusions with a net anterograde movement at a speed consistent with myosin motor-dependent transport [87]. Proteins localized to these projections include Cofilin and Myosin-X, and these are seen to travel towards and accumulate at the distal tip of these protrusions. Stabilised interactions are formed between mesenchymal cell protrusions and additional protrusions emanating from the receiving cells that contain a subset of Shh co-receptors. Both Cell adhesion molecule Downregulated by Oncogenes (Cdo) and Brother of Cdo (Boc) co-localize in specific microdomains within responding-cell filopodia [87]. Here, filopodia-like protrusions provide a means for long-range morphogen movement without the need for free diffusion.

Transport of Wnts by cytoplasmic filopodia has also been observed in multiple situations. During patterning of the zebrafish neural ectoderm, Wnt8a, produced at the blastoderm margin, acts as a posteriorising factor for the distant midbrain–hindbrain boundary [88]. Live imaging of fluorescently tagged Wnt8a in zebrafish embryos revealed its localization to membrane-associated punctae within filopodia-like protrusions. Clusters of Wnt8a and the receptor Frizzled were identified on responding cells that were proposed to derive from Wnt8a transported on the protrusions [89]. In addition, cells of the gastrula neural plate were also recently reported to transport Wnt8a along short (~10–50 µm) actin-based filopodia, moving toward the distal tips away from secreting cells [90]. Filopodia contact a neighbouring cell where they activate Wnt signaling, with the Wnt8a positive tips observed to form extracellular punctae. Wnt8a positive filopodia formation is dependent on Cdc42 function. Blocking filopodia formation, through the overexpression of a mutated form of the Cdc42 effector IRSp53, causes posterior expansion of the anterior brain structures similar to phenotypes observed when Wnt antagonists are activated. Levels of Wnt target gene production were not affected by filopodia formation, but the range of Wnt signaling was found to be correlated with length and filopodia number, with inhibition of filopodia formation resulting in a shorter signaling range and steeper gradient. These data demonstrate the importance of this short-range filopodia-based transport for patterning of the neural plate during gastrulation by increasing the effective Wnt8a signaling range [90].

## Packaging ligands into extracellular vesicles

Some extracellular ligands appear paradoxically ill-suited to free extracellular diffusion due to post-translational lipid-modifications that drive their membrane association. Therefore, one mechanism for transporting lipophilic ligands is to load them onto lipid-based transporters.

It was first suggested that lipophilic ligands could ‘hitch a ride’ on and/or within extracellular membranous vesicles resembling exosomes that had originally been described in haematopoietic cellular communication [91]. Exosomes are small 40–100 nm vesicles composed of a lipid bilayer that are produced within multivesicular bodies (MVBs) that fuse with the plasma membrane to release the vesicles [92–94]. It was shown that GPI-anchored GFP expressed in the *Drosophila* wing disc migrated away from the expressing cells in small particles. Labelling both the inner and outer membrane leaflets revealed that these particles, termed argosomes, were composed of a membrane bilayer and when GPI-GFP expression was driven in Wg-expressing cells, Wg was found to co-localize within these migrating GFP positive particles [91]. Argosomes were, therefore, proposed to act as a vehicle for the diffusion of Wg.

Members of the Wnt protein family, with the exception of *Drosophila* WntD [95, 96], undergo both palmitoylation and N-glycosylation [96–98]. The former in particular, mediated by the membrane-bound O-acyltransferase Porcupine (Porc), is fundamental to Wnt signaling [96, 99, 100] as it is essential for Wnt recognition by the transmembrane carrier protein Wntless (Wls) for trafficking through the endocytic pathway and eventual secretion [101–104]. In addition, the absence of palmitoylation has been shown to weaken ligand-receptor binding [101, 105, 106] and the crystal structure of *Xenopus* Wnt8 in complex with murine FZD8 reveals that one of the two binding interfaces is dominated by a palmitoleate moiety and a hydrophobic groove on the FZD8 cysteine-rich domain [107]. Lipidation is, therefore, essential for functional Wg and extracellular vesicles may act to promote ligand diffusion by protecting/hiding the lipid moiety.

Similar structures were later identified in the *Drosophila* nervous system and in cell culture. Wg and Wls are trafficked between synapses or cells in exosome-like vesicles [108]. Electron microscopy (EM) revealed their initial intracellular localization within MVBs, while mass spectrometric analysis of S2 cell-derived Wls^+^ vesicles revealed that they contain many key classes of exosome associated proteins, including membrane trafficking components (Annexins, Rho proteins), V-ATPase subunits and proteins involved in lipid raft (Flotillin-1) and MVB formation (Alix, Clathrin) [109].

A number of studies have now revealed the conserved role of exosomes in Wnt signaling. Human, mouse and *Drosophila* cells have been shown to secrete exosome-like vesicular structures that contain exosomal markers, such as human CD63 and murine TSG101. A portion of Wnt3A, in murine fibroblast cells, and Wnt5A, in human Caco-2 cells was found to co-fractionate with their respective exosomal markers and EM analysis of *Drosophila* Kc167 cell culture supernatant reveals small vesicles (40–100 nm) with Wg localized to the outer surface. Importantly, the exosome-bound pool of Wnts is biologically relevant as they are able to activate Wnt responsive reporters in cell culture [110, 111].

As was seen in the *Drosophila* nervous system, Wls-GFP also co-localizes with Wnts and exosomal markers within MVBs in the wing disc and the culture media of HEK293 cells and S2 cells suggesting a role in the shuttling of Wnts in these intraluminal vesicles. However, Wls is not found to significantly co-localize with Wg on exosomes from the wing imaginal disc, suggesting that Wls is dispensable following trafficking to the MVB [111]. The small GTPase Rab11 is necessary for Wg-exosomal secretion in S2 cells [111], as had been previously shown in the *Drosophila* larval neuromuscular junction where *Rab11* RNAi knockdown depletes the Wg-exosomal localization without affecting its overall secretion [109]. In vivo depletion of Rab11 in the wing imaginal disc leads to the apical accumulation of Wg puncta suggesting perturbed intracellular trafficking; however, this had no effect on the distribution of extracellular Wg and gradient formation. Therefore, while Wg is secreted on exosomes in the wing disc, this may not be necessary for Wg secretion and gradient formation in this context. It was not, however, directly shown that exosome secretion was indeed blocked upon Rab11 depletion and it remains a challenge investigating the role of exosomes in vivo in the absence of a complete understanding of exosome biology and the pleiotropic nature of endosomal trafficking disruption. Nonetheless, the possibility also remains open for roles of exosomes in Wnt signaling in other systems.

Another morphogen was also identified around the same time as Wg to use this cellular shuttle bus service. The Hh family is lipid-modified through the addition of N-terminal cholesterol and C-terminal palmitic acid, both of which are necessary for controlling extracellular diffusion and signaling in a number of contexts [112]. In the *Drosophila* embryo and wing imaginal disc, cholesterol-modified Hh is largely localized to the plasma membrane of producing cells, although it does also move away [113, 114]. In the embryo, Hh localizes to cytosolic and plasma membrane-associated puncta in a cholesterol-dependent manner [114, 115]. GPI-anchored Hh, which was not cholesterol modified, was unable to move and instead remained associated with the producing cell’s basolateral membrane which significantly reduced signaling range. Therefore, Hh association with migrating puncta is dependent on cholesterol and not simply membrane anchoring [115].

The nature of these plasma membrane-associated puncta was later clarified by multiple studies in diverse biological contexts. Shh and retinoic acid were found to associate with similar punctate structures, termed ‘nodal vesicular parcels’, secreted by cells of the chick node. These extracellular vesicles associate with microvilli and migrate along the stream of the nodal flow to transport Shh and retinoic acid during left–right patterning [116]. Again, similar MVB-derived structures were identified in the epidermis of *C. elegans* during cuticle formation [117]. Perturbed exosome release from MVBs, through mutation of *vha*-*5* (a V-ATPase subunit), drives severe cuticle malformation. Collagen secretion was found to be unaffected; however, the secretion of the GFP-tagged Hh-related peptides, WRT-2 and -8 [118], was inhibited and the ligands accumulated within MVBs. Furthermore, mutation of *che*-*4*, the *dispatched* (*disp*) homologue, partially resembles the abnormal cuticle phenotype observed in *vha*-*5* mutants [117]. Disp is a regulator of Hh protein secretion and its loss of function inhibits the apical release of Hh in the *Drosophila* ectodermal epithelium [115, 119]. Together these studies suggested a novel pathway for Hh ligand secretion and extracellular transport through the release of MVB-derived extracellular vesicles.

**Fig. 4:**
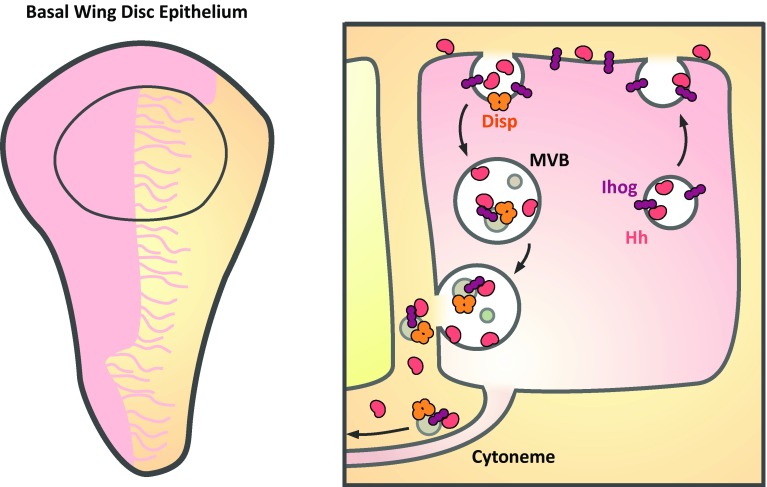
Hh hitches a ride on exosomes that travel along cytonemes in the *Drosophila* wing disc. *Left panel* Hh producing cells (*pink*) of the wing disc epithelium produce cytonemes that extend over the domain of Hh signaling. *Right* Hh and its co-receptor Ihog are initially secreted apically before being endocytosed in a Disp and Rab5-dependent manner. Re-internalised Hh, Ihog, and Disp undergo transcytosis and are secreted basally; some are found within MVBs on intraluminal vesicles (*grey*). MVBs fuse with the basal membrane and release exosome-bound Hh, Ihog, and Disp that travel along cytonemes

Recent work in the *Drosophila* wing disc now suggests a role for these MVB-derived extracellular vesicles in long-range Hh signaling. Cells of the posterior wing disc secrete Hh that localizes to basal cytonemes with its co-receptor Interference hedgehog (Ihog) in punctate structures (0.2–0.6 µm) that exceed the diameter of the cytonemes [120–122] (Fig. 4). The cytonemes extend 7–12 cell diameters (up to 70 µm) into the anterior compartment, covering the majority of the Hh signaling gradient and could, therefore, play a key role in Hh gradient formation. Time-lapse imaging reveals that the puncta migrate along cytonemes away from producing cells [123], similar to SHH puncta in the chick limb bud that travel along specialized actin-based filopodial extensions [87]. EM imaging of Hh and Ihog localization reveals discrete basolateral localization within MVBs and on vesicle-like structures (0.03–0.2 µm) within the extracellular space in close contact with cellular protrusions [123]. Moreover, a portion of Hh and Ihog co-localizes with the ectopically expressed exosomal marker CD63 and secreted Hh from insect cell media co-fractionates with exosome-associated factors, including TSG101, Rab11, Rab8, Syntaxin and Hsp70 [123, 124]. Importantly, this vesicle-associated pool of Hh is signaling competent as fractionated exosomes are able to induce both a *ptc* reporter and phosphorylation of Fused, while in vivo RNAi against exosome associated proteins, such as Rab11 and AnxB11, all reduce the length of the Hh signaling gradient [122–124]. Much still remains unclear, however, particularly how these ligands are loaded onto morphogens.

Disp has long been suggested to regulate the release and long-range signaling of Hh ligands [119, 125–127]. In the absence of Disp function Hh synthesis, lipid modification and apical exocytosis in the wing disc are unaffected. However, Hh accumulates at the apical plasma membrane and only juxtacrine signaling is maintained [119]. Endocytosed Hh co-localizes with Disp and Rab5 in producing cells and in the absence of Disp or Rab5 activity, lipid-modified Hh is no longer endocytosed or accumulates in early endosomes, respectively. It has therefore been suggested that Disp ‘captures’ apical Hh and drives its trafficking through the endocytic pathway where it is transcytosed to the basolateral membrane [124] (Fig. 4). A similar mechanism was also recently described in Wg signaling. In the wing disc, Wg displays distinct apicobasal localization, with intracellular Wg accumulating apically and extracellular Wg largely found basally. By tracking the progression of newly synthesized tagged-Wg proteins, it was shown that Wg is first apically trafficked before being transcytosed to the basal membrane [128] where it is secreted [129]. This was found to be regulated by the E3 ubiquitin ligase Godzilla (Gzi), which was previously shown to regulate recycling endosome trafficking [130]. Disruption of *gzi* expression or activity results in Wg apical accumulation and the loss of target gene expression showing that transcytosis is necessary for Wg signaling. Indeed, the Wg receptor Fz2 is also basally enriched. It is suggested that this convoluted secretory pathway may be necessary for ligand maturation and/or for circumventing contact between Wg and Notum, an inhibitor and target gene of Wg, during secretion [128]. Although not investigated in these studies, this ligand trafficking could potentially play a role in the exosomal loading of ligands.

Ubiquitylation provides a common route for the sorting of cargo to early endosomes that mature into MVBs through the ESCRT-mediated formation of intraluminal vesicles [131]. In the case of Hh, it can be found in endocytic vesicles along with Ihog and Disp travelling to the basolateral membrane as well as on intraluminal vesicles within MVBs [120, 123, 132]. While the role of MVBs in Hh-associated exosome formation is debated, the formation of a portion of these exosomes has been suggested to be dependent on ESCRT machinery [124, 132] and in vivo knockdown of ESCRT complex protein expression reduces Hh secretion and signaling [124]. Conversely, other alternative mechanisms could drive the process or work in conjunction with this; for example, knockdown of Sphingomyelinase, a regulator of ESCRT-independent exosome formation, in the wing disc similarly reduces Hh secretion [123].

The overall need for exosomes in Wg and Hh signaling remains up for debate and further investigation is made difficult by the problems associated with manipulating fundamental cellular machinery. It is clear that exosomes are not working alone, with additional carrier proteins also acting to promote ligand diffusion. In *Drosophila*, the lipocalin family member, Swim, binds to and shields the lipid moiety of Wg and is necessary for long-range signaling in the wing disc [133]. Similarly, the secreted HSPG Carrier of Wg (Cow) [134] and vertebrate secreted Fz-related proteins (sFRPs) [135] have been shown to support ligand diffusion. In zebrafish and mammalian cell culture, the diffusion of Hh ligands is promoted by Scube family members [136, 137]. The cholesterol-dependent binding of Scube2 has been shown to link HSPG-associated Shh to proteases that process it for release from the cell membrane and enhance ligand solubility through shielding of the cholesterol moiety [138–140]. Both Wg and Hh have also been found associated with Lipophorin in lipoprotein particles in the *Drosophila* wing disc [141, 142].

## Long-range action revisited

While recent studies have highlighted new strategies for controlling signaling range, methodological advances have also allowed the requirement for long-range signaling to be re-evaluated in certain contexts, for example Wg signaling in the *Drosophila* wing disc. Evidence supporting the long-range action of *Drosophila* Wg in the wing disc came from the identification of nested expression domains of different target genes, analysis of loss-of-function clones at a distance from the source cells, as well as the results obtained from misexpression of either wild type Wg or Nrt-Wg, a membrane-tethered form [143, 144]. While misexpression of Wg was found to activate target genes up to many cell diameters away from the clones of expressing cells, in contrast, Nrt-Wg only activated target genes in expressing and immediate neighbour cells [144]. In addition, Wg protein was visualized in a gradient in the wing disc many cell diameters away from the expressing cells at the DV boundary [129, 143, 145].

However, recently the requirement for Wg spreading has been revisited by an alternative experimental approach that exploited advances in genome engineering to replace the endogenous *wg* gene with the membrane-tethered form, *Nrt*-*Wg* [146]. Surprisingly, flies homozygous for the *Nrt*-*Wg* allele appear wild type except for a short delay in developmental progression and marginally smaller wings. While Wg target gene expression in the wing imaginal discs was mildly altered by the loss of gradient formation, many key genes were still found to be expressed in broad patterns away from the Wg-expressing cells during late larval development. Temporal analysis of endogenous *wg* transcription suggests that *wg* is initially active in cells throughout the wing disc pouch before becoming restricted to a narrow stripe of cells along the dorsal–ventral compartment boundary. As target genes are still expressed in a broad pattern in response to the stripe of tethered Wg, it has been proposed that this low-level pre-pattern and transcriptional memory of earlier signaling may enable the perdurance of Wg target gene expression during wing disc development, in the absence of diffused Wg in regions far from the dorsal–ventral boundary. Based on these observations, it was concluded that although there is evidence for a long-range Wg gradient, Wg spreading is instead largely dispensable for complete organismal patterning and growth regulation [146].

The role of the classical wing disc Dpp gradient has also been revisited by genome engineering. Many studies support this Dpp gradient, emanating from a stripe of *dpp* expressed at the anterior–posterior compartment boundary, directing growth and patterning of the wing [147]. Genome engineering was used to introduce FRT sites flanking the first *dpp* coding exon, to allow Flp recombinase mediated removal of this exon in particular cells and/or at a specific time. Surprisingly, specific removal of *dpp* expression from the stripe at the anterior–posterior (AP) compartment boundary, by *dpp* enhancer driven expression of the Flp recombinase, revealed only minor growth defects in third instar larvae. Evidence is presented that growth is instead supported by earlier *dpp* expression in the anterior compartment. However, Dpp target gene expression was lost upon removal of the anterior–posterior stripe of *dpp* expression, suggesting that the Dpp gradient is required for patterning but not growth [148].

A separate study manipulated Dpp–GFP spreading in the wing disc through the localized expression of an anti-GFP nanobody, a single domain camelid antibody, which immobilizes Dpp–GFP. Results from this study indicate that Dpp spreading is required for cell patterning [149], consistent with the results observed upon removal of *dpp* stripe expression [148]. However, preventing Dpp–GFP from spreading reduced cellular growth in the medial but not lateral regions of the wing disc [149], in contrast to the *dpp* exon deletion experiments that found the stripe of expression to be dispensable for growth [148]. Further experiments are required to resolve this discrepancy, but one possibility may be that there is transient expression of *dpp* in the AP stripe (prior to Flp-mediated removal of the essential exon) that is sufficient to promote growth in the medial region.

## Perspectives/outlook

The different strategies used to control signaling range that we have described here highlight how few rely on passive diffusion of the signaling molecule. A classic example of free diffusion is the formation of the Bicoid gradient in the *Drosophila* embryo. Here, maternally deposited mRNA is anteriorly localized and Bicoid protein diffuses within the syncytial cytoplasm from this source generating an anterior–posterior gradient [150]. Similarly, the majority of fluorescently tagged Fgf8 in zebrafish embryos undergoes rapid, free diffusion, and a gradient is established through an endocytosis-dependent source-sink mechanism [151, 152]. However, while this simple mechanism is attractive, the reality seems to be that diffusion of signals is often limited by receptors, the extracellular matrix, and/or tissue architecture.

Given that diffusion is likely to only be appropriate in limited contexts, cells have adapted to increasing organismal complexity by evolving specialized structures and proteins that facilitate ligand movement and drive long-range signaling. Indeed, membranous protrusions and extracellular vesicles are highly conserved phenomena that have been shown to drive lipophilic ligand diffusion in a variety of contexts. Given the multitude of roles that signaling ligands play in development and tissue homeostasis, it is possible that these methods of active morphogen movement may represent common mechanisms for driving long-range movement, even if long-range signaling may in fact be dispensable in some contexts. However, the individual contribution of membranous protrusions or extracellular vesicles to cell fate patterning remains unclear, due to the potentially pleiotropic nature of the methods used to manipulate these structures to date. Advances in this area will rely on determining how these structures are formed, how ligands are targeted to them, and how they actively drive movement, to manipulate each specific mechanism.

In terms of future advances, genome engineering is likely to make a big impact, as for the study of *Drosophila wg,* given the opportunities it affords for manipulating endogenous signaling molecules with precise spatial and temporal control [146]. Similarly, nanobodies offer new strategies for manipulating signals. In particular, the anti-GFP nanobody can be used to target GFP fusion proteins, including GFP protein traps, for degradation or immobilization in a particular domain [149, 153]. In addition, nanobodies have been raised that are specific to either the inactive or active conformations of the EGF receptor [154]. Conformation-specific nanobodies will be valuable tools for probing different aspects of cell signaling, including potentially the specific detection of signals bound to extracellular antagonists compared to the unbound pool, for example, as antagonist binding is often associated with a conformational change in the signaling molecule [155]. Despite these advances, the inability to routinely visualize signaling molecules still hampers their study, especially given the opportunities offered by super resolution microscopy. Perhaps the highly antigenic ‘spaghetti monster’ fluorescent proteins (smFPs) that allow enhanced detection of low abundance proteins [156] will aid visualization of extracellular signals.
